# Radiomics-Guided Precision Medicine Approaches for Colorectal Cancer

**DOI:** 10.3389/fonc.2022.872656

**Published:** 2022-06-09

**Authors:** Mohammed I. Quraishi

**Affiliations:** Department of Radiology, University of Tennessee Medical Center, Knoxville, TN, United States

**Keywords:** radiomics, colorectal, cancer, precision medicine, personalized medicine, precision oncology, genomics

## Abstract

The concept of precision oncology entails molecular profiling of tumors to guide therapeutic interventions. Genomic testing through next-generation sequencing (NGS) molecular analysis provides the basis of such highly targeted therapeutics in oncology. As radiomic analysis delivers an array of structural and functional imaging-based biomarkers that depict these molecular mechanisms and correlate with key genetic alterations related to cancers. There is an opportunity to synergize these two big-data approaches to determine the molecular guidance for precision therapeutics. Colorectal cancer is one such disease whose therapeutic management is being guided by genetic and genomic analyses. We review the rationale and utility of radiomics as a combinative strategy for these approaches in the management of colorectal cancer.

## Review

### What is Precision Medicine/Oncology?

In the early part of this century, the term *personalized medicine* was heavily used to promote a new paradigm of treatment tailored to an individual. However, as this field developed, this term was seen as a misnomer as it highlights the individual and not the disease process. The term *precision* medicine more aptly describes how treatment is tailored to tumor-specific features that can be shared between individuals (1). Features of a disease state can be found in genetic, imaging, and histological information, and have been explored using deep analytics with genomics, radiomics, and pathomics, respectively.

Precision oncology, the application of this paradigm to oncology, has potential to revolutionize cancer management. This is a developing field with robust ongoing research. The greatest strides have been in genomics with radiomics not far behind.

### The Genetics/Genomics Landscape of Colorectal Cancer

The theory of genomics in precision oncology is that treatment can be tailored based on the genetic make-up of the cancer (2). The most suitable therapy can be selected either by correlating between the unique genetic fingerprint of the malignancy and therapy options, or by impeding the driver mutation of an identified specific oncogene.

There is robust research in this field mainly due to recent availability and affordability of high-quality NGS molecular analysis (3). An example of that is the HER2 positivity, a clinically relevant genomic marker for breast cancer that predicts response to trastuzumab-based therapies (3). Trastuzumab binds to the HER2 receptor which results in inactivation of the intracellular tyrosine kinase and, therefore, handicaps cell proliferation (4). Lung cancer management has also been more recently transformed by precision oncology approaches. Currently, instead of treating all patients with platinum-based doublets, treatment takes into consideration genetically defined subsets. For example, PD-L1 expression denotes a stronger response to immunotherapy (3). More recently, genomic subtyping has been shown to be prognostic in pulmonary large-cell neuroendocrine carcinoma (5).

The advent of NGS has made finding mutations in colorectal cancer more applicable to clinical practice. Genetic testing in colorectal cancer for prognostication and therapy selection is now standard of practice. In EGFR-expressing colorectal cancer (CRC), the mainstay of treatment is EGFR-targeting antibodies. However, only a limited percentage of CRC with EGFR expression respond. Currently, prognostication of treatment response with anti-EGFR correlates with specific KRAS, NRAS, and to a lesser extent BRAF V600E mutations. These mutations predict poor treatment response to anti-EGFR treatment. The presence of the KRAS or NRAS mutation confers not only poor response but also shorter survival if anti-EGFR treatment is used (6).

Beyond anti-EGFR therapy, newer therapeutic approaches are increasingly being guided by genetic/genomic advancements. Clinical trials studying the targeting of the BRAF V600E mutation with triple therapy (anti-RAF, anti-MEK, and cetuximab) are showing promise (6). Amplification of HER2 (ERBB2) also predicts poor anti-EGFR response as seen in the HERACLES trial (6, 7). Fusion involving the NTRK family of genes are seen in some CRC. Anti-NTRK treatment has shown promise in this subtype of CRC. Further, fusion in ALK, ROS, and RET all are present in different subtypes of CRC and show promise with targeted therapies (6).

Tissue heterogeneity is the main culprit in poor clinical adoption of new genomic insights for colorectal cancer. Within the same tumor there can be heterogeneous genetics that can be missed due to biopsy sampling error. Tissue heterogeneity can also be seen in colorectal cancer between the primary site and metastatic sites. Even more challenging are the changes in the genetic make-up of the disease seen after therapy. Furthermore, it is practically difficult to subject the patient to repeated biopsies for tissue/molecular/genetic analyses (6). It can be postulated that these issues can be allayed by radiomics. The phenotypic heterogeneity of tissue and its metastatic foci can be deciphered from imaging data. Moreover, imaging is easily repeatable, making it feasible to continually assess radiomic endpoints that represent intratumoral patterns of therapy response. In a synergistic fashion, radiogenomics, we believe, will enable precision oncology to deliver at its highest potential.

### Radiomics as a Non-Invasive Imaging Correlate to Pathophysiologic and Genetic Basis of CRC

As a quantitative image analysis technique, radiomic analysis yields signatures (set of features) that may be shown to correlate with molecular processes or structural changes implicated in tumor behavior and its responsiveness to therapy. A wide range of radiomic signatures have been extracted in a number of studies focusing on CRC and were found to be useful in improving the way CRC is classified (8), stratified (9), and assessed for their natural history and therapy response (10). Radiomics is also improving our ability to develop more effective therapies to fight cancer by way of providing endpoints for pharmacokinetic/pharmacodynamic assessment as well as early therapy assessment response (or even predictive) biomarkers.

The emerging landscape of radiomics in CRC includes applications of this technique for the improved classification of CRC. Studies have shown that CT-based radiomic signatures were able to distinguish between high-grade and low-grade (AUC 0.7; 0.9) (11), and between the stage I-II and stage III-IV of CRC (AUC 0.8) (12), and for the prediction of microsatellite instability (MSI) in CRC which has implications for therapy responsiveness. Radiomics should be seen as a complimentary approach (and not as an alternative) to standard clinicopathologic methods used to guide clinical management. In a recent randomized controlled trial, radiomics was performed on preoperative CT in stage II and III CRC and it was observed that the combined clinical-radiomics model predicted the preoperative MSI status more accurately than the clinical or radiomic models alone (AUC 0.8) (13).

Radiomics has the potential to noninvasively predict genetic mutations/alterations which have implications in the personalized management of CRC. In a study by Tien et al., it was observed that CT-based radiomics predicted the KRAS/NRAS/BRAF mutations while clinical background, tumor stage and histological grade had no significant association (AUC 0.8) (14). FDG-PET-based radiomics analysis has also been used to determine various genetic alterations in CRC. Kao et al. observed that CRC with a mutated KRAS showed a 25th-percentile increase in the standardized uptake value (SUV) in their metabolic tumor volume (MTV) (15). It was also shown that the mutated TP53 was associated with an increased value of short-run low gray-level emphasis derived from the gray-level run length matrix, while the APC mutants exhibited lower low gray-level zone emphasis derived from the gray-level zone length matrix (15). In another study, CT-based radiomic analysis that quantified the temporal decrease in the tumor spatial heterogeneity and boundary infiltration were found to better predict the sensitivity EGFR-targeting therapy in metastatic CRC as compared to the standard endpoints, such as KRAS mutations and tumor shrinkage as per RECIST 1.1 (16)​.

More sophisticated radiomic analyses are possible with MRI, a modality used regularly in the management of CRC. A 30-feature radiomic signature extracted from pre- and post-therapy MRI (along with tumor length) was shown to predict the pathologic complete response to locally advanced CRC (AUC 0.98) (17). Another study showed that T2W-MRI based radiomics was superior to T2W/DWI at predicting pathologic complete response to neoadjuvant therapy for CRC (AUC 0.93) (18). FDG-PET based radiomics has also been found to be useful for therapy response prediction. In a recent study, it was shown that the radiomics-based tumor heterogeneity along with low tumor volume, both measured on FDG PET/CT were associated with improved clinical outcome (AUC 0.8) (19).​ A combined multiparametric approach, using FDG-PET and MR-based radiomics assessment was found highly predictive of neoadjuvant therapy response (20).

### ​Combining Radiomics With Genomics for Precision Therapy Guidance for CRC

As shown by several studies mentioned above, radiomic biomarkers can be correlated with and potentially predict genetic mutations on one hand and clinical outcomes on the other. Furthermore, it can be proven to be a highly useful methodology when combined with genetic/genomic studies to understand the complete genotype-phenotype relationship that is implicated in the natural history and therapy responsiveness of CRC. Such radiogenomic methodologies have been employed in diseases, including Non-small lung and breast cancers (21) and utilizing it may be of great benefit in the precision management of CRC as well.

**Figure 1:**
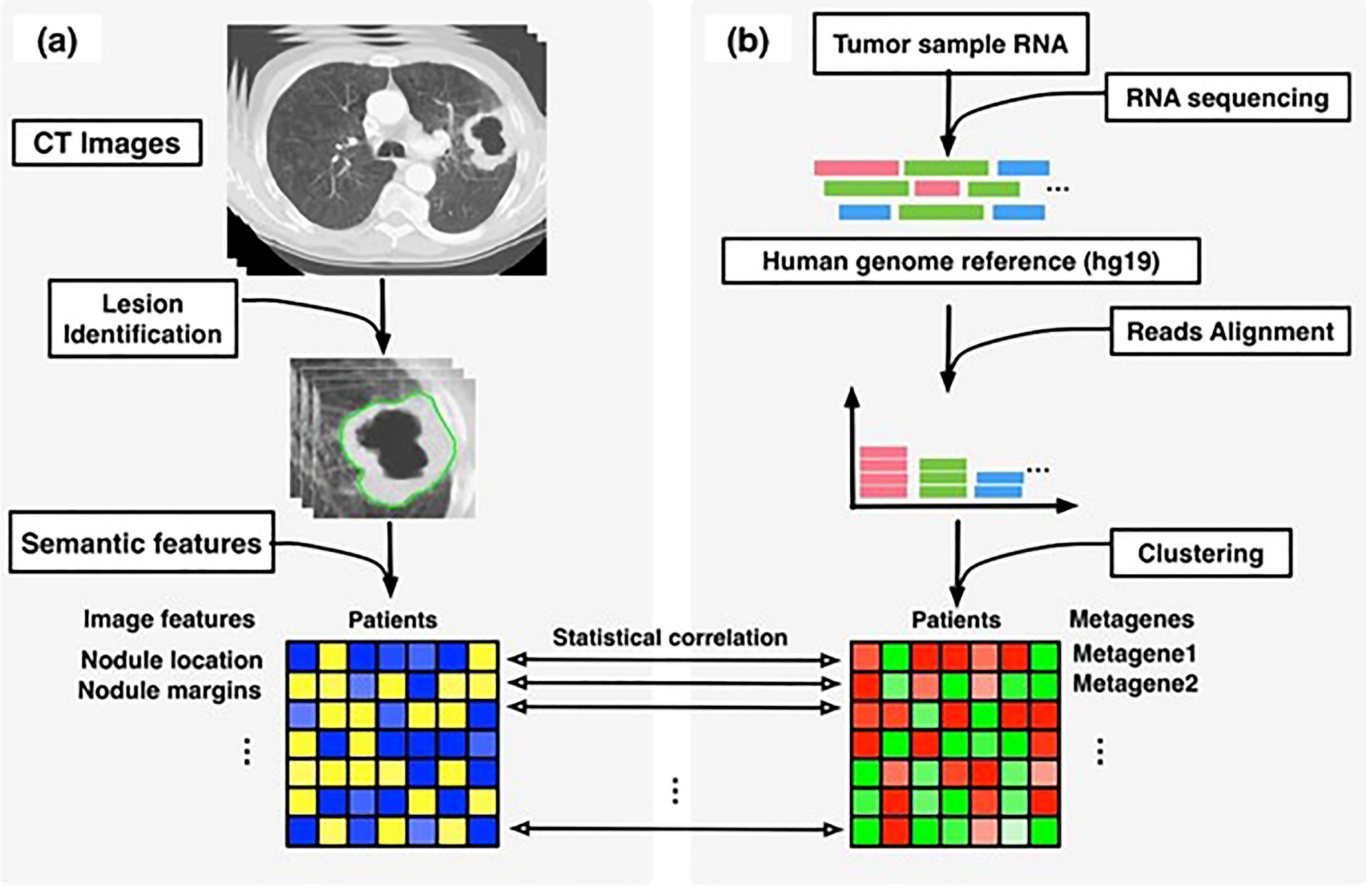
Overview of radiogenomic analysis to identify associations between, **(A)** semantic features at CT and, **(B)** RNA sequencing data. [reused from Zhou et al. (24) under the CC-BY license].

This combinative multi-omics approach has been used to evaluate the pathophysiologic underpinnings and the natural history they impact (22, 23). Radiogenomics involves extracting radiomic features from a CT, MR or a PET scan and combining it with the mRNA expression data to create a radiogenomic signature and correlating it with the clinicopathological event under study (see **Figure 1**). This was performed in a study performed by Duo et al, in which they employed a radiogenomic approach using FDG-PET/CT and messenger RNA data to develop a signature that depicted the epithelial–mesenchymal transition in non–small cell lung cancer (25). Such methodologies have been attempted in the realm of CRC as well. Bogdan et al. studied the combined signature from CT and ABC22, CD166, CDKNV1 and IHBBB gene expression and histologic grading to be an statistically significant prognosticator for CRC screening and management (26).

Studies have been performed to study the genetic changes and the radiomics features and if they can be used to guide precision therapy. Lambin et al. have demonstrated that radiomics can identify the gene expressions related to tumor cells in response to doxycycline and radiation treatment (27). Radiomic signatures that can predict the V-Ki-ras2 Kirsten rat sarcoma viral oncogene homolog (KRAS) mutation in CRC have been studied, where it was interesting to note that radiomic signal based on CT was superior to FDG PET for that purpose (28). In a retrospective study performed by Petskova et al., a radiogenomic analysis using the genomic sequences and the pretreatment MRI was performed, which showed that quantitative assessment (radiomics as opposed to qualitative assessment) showed an association with genetic mutation related to CRC (29).

These approaches, while potentially highly beneficial are not without their challenges. The robustness of radiomics/radiogenomics in terms of inter- and/or intra-reader agreement continues to be scrutinized. Having said that, many studies have concluded that these techniques are reliable in that regard, especially when the models have large datasets to build on and the segmentation capabilities improved (which can be challenging in CRC lesions) (30).

### Other Considerations

Here we have focused on the emerging role of radiogenomics for colorectal cancer as we believe it will be a pillar of precision oncology. However, it should be noted that beyond imaging biomarkers and genetic make-up, environmental and epidemiological factors also affect cancer behavior. Varied factors such as alcohol intake, processed meat consumption, gut flora makeup, cigarette smoking, infanthood bottle feeding, sedentary lifestyle, and obesity amongst others are implicated in cellular epigenetic and genetic alterations which can predispose an individual to CRC (31). This is termed the exposome, which describes the sum of exposures and their interactions (31). The field of molecular pathological epidemiology (MPE) looks at how epidemiologic factors affect molecular pathology, and as MPE develops there is potential for finding exposome biomarkers (32). Studying the interplay between imaging, genetics, pathology, and the exposome to develop a more precise tumor signature is the terminus of precision oncology.

## Conclusion

Precision management of CRC is poised to benefit from the noninvasive radiomic correlates to the genetic information implicated in its driving decision making process as well as the combined radiogenomics approaches that study the genotype-phenotype relationship at its core.

## Author Contributions

The author confirms being the sole contributor of this work and has approved it for publication.

## Conflict of Interest

The author declares that the research was conducted in the absence of any commercial or financial relationships that could be construed as a potential conflict of interest.

## Publisher’s Note

All claims expressed in this article are solely those of the authors and do not necessarily represent those of their affiliated organizations, or those of the publisher, the editors and the reviewers. Any product that may be evaluated in this article, or claim that may be made by its manufacturer, is not guaranteed or endorsed by the publisher.
